# Effective Adoption of the Supportive Coordinated Transitional Care Program Improves End-Of-Life Care For Veterans

**DOI:** 10.1093/geroni/igaf122.2610

**Published:** 2025-12-31

**Authors:** Lana Brown, Hallie Keller, Tanya Clayton, TaFarra Haney, Ramona Rhodes, Wanda Morrison, Jane Driver

**Affiliations:** Central Arkansas Veterans Healthcare System, North Little Rock, Arkansas, United States; Central Arkansas Veterans Healthcare System, North Little Rock, Arkansas, United States; Central Arkansas Veterans Healthcare System, North Little Rock, Arkansas, United States; Eugene J. Towbin Healthcare Center, North Little Rock, Arkansas, United States; UT Southwestern Medical Center, Dallas, Texas, United States; Central Arkansas Veterans Healthcare System, North Little Rock, Arkansas, United States; VA Boston Healthcare System, Brockton, Massachusetts, United States

## Abstract

The Coordinated Transitional Care (CTrac) program is a telephone-based, nurse-driven program shown to decrease hospital readmissions in acute care hospitals. This project implemented and evaluated an adapted version of CTraC, Supportive CTraC, developed at the Veteran Affairs (VA) Boston, to enhance the quality of transitional and end-of-life care for veterans with advanced disease and a life expectancy of ≤ 24 months. The Replicating Effective Programs framework, led by two registered nurse case managers, was used to adapt and implement the Supportive CTraC program at one VA facility. Program success was defined as > 30% of participating Veterans referred to palliative care/hospice, >30% enrolled in hospice, >30% with new Advance Directives, and fewer acute care deaths compared to the hospital average. Two-hundred three Veterans aged 43 to 96 enrolled in the program. Veterans were primarily male (96%), White (61%) or Black (34%), and non-Hispanic (97%). About half (52%) were urban residents compared to rural (42%) or highly rural (5%). During the 8-week program, greater than 30% of Veterans completed an Advance Directive (57%), received a hospice/palliative care consult (48%), and enrolled in hospice (39%). By the end of the program, 135 (67%) Veterans had died. Of these, fewer died in acute care compared to the hospital average (20% vs. 36% ). The remaining Veterans died at home (41%), in hospice/palliative care (36%), a nursing home or rehabilitation unit (2%), or an unknown setting (1%). The Supportive C-TraC program was successfully adapted and implemented, meeting all goals and facilitating positive end-of-life outcomes for Veterans.

